# Disaster preparedness training for tribal leaders

**DOI:** 10.1186/1745-6673-3-2

**Published:** 2008-01-15

**Authors:** Wayne F Peate, Jennie Mullins

**Affiliations:** 1 Mel and Enid Zuckerman Arizona College of Public Health, University of Arizona, Tucson, Arizona, USA

## Abstract

It was with considerable irony that tribal leaders began a collaboration with the University of Arizona and the Arizona Department of Health Services for training in public health preparedness, as the tribes had an extended prior history of responding to a host of hazards caused by the dominant culture. The objective of the training was to ensure that Native American communities were adequately informed and trained to implement coordinated response plans for a range of potential public health emergencies on tribal lands and in surrounding communities. This commentary outlines how cultural competency (including public prayer by an elder during the training), respect for tribal sovereignity, solicitation of historical examples of indigenous preparedness, and incorporation of tribal community networks were essential to the success of this program.

Tribal Public Health Preparedness and Response: Homeland Security Since 1492

## Background

A popular tee-shirt in Native-American tribal communities portrays a photo from the 1880s of Geronimo, the last tribal leader to lead active armed resistance against the Federal Government, with the caption "Homeland Security Since 1492." It was with considerable irony, therefore, that tribal leaders began a collaboration with the University of Arizona and the Arizona Department of Health Services for assistance in training for public health preparedness, as the tribes had an extended prior history of confronting and responding to a host of hazards caused by the dominant culture.

The approach was to be "all hazard," including: preparation for fire (Arizona has been devastated by the loss of 800,000 acres from wildland fires in the past three years); floods from the Colorado River and other bodies of water; and infectious diseases such as West Nile Virus, Avian Influenza and other bioterrorism agents [1].

When the discussions led to preparation for these events, tribal members offered poignant reminders of the public health consequences of European domination, including:

Biologicals: the loss of entire communities to smallpox and other infectious agents

Fire: Tribal practice had wisely permitted wildland fires as part of a natural process. In contrast, federal policy for years had been zero tolerance for any fire. As a result, thick underbrush had choked many forests and led to a dangerous build up of fire fuels that led to uncontrolled conflagrations.

### Competency based

Cultural competency. Each training session began and ended with a prayer lead by member of the host tribe. Separation of religion and state was respected, but so was the cultural heritage of the participants. "The Whites talk of mind, body and spirit. For us everything is spiritual from the time we get up until we go to bed" (personal communication Annie Kahn, Navajo healer). Prayers focused on themes such as "protecting the people," the concept of health as not only physically, but also mental well-being, and the encouragement of cooperation between all humanity, no matter their ethnicity.

### Objectives

The overall objective of this project was to provide basic public health emergency and bioterrorism preparedness and response training for tribal personnel through developing and delivering three training modules and coordinating implementation statewide by means of five regional 1 1/2 day sessions in close cooperation with the Arizona Department of Health Services (ADHS), Office of Public Health and Emergency Preparedness Response (OPHEPR) and its Native American Liaison.

The purpose of providing such training was to ensure that Native American communities were adequately informed, aware and skilled to implement coordinated response plans for a range of potential public health emergencies on tribal lands and in surrounding communities. Training of tribal public health professionals, emergency management personnel and health care system providers, as well as representatives of tribal community networks, was considered a priority to strengthen the tribes public health emergency preparedness infrastructure.

### Target audience

The initial target audience was personnel from the 12 Arizona tribes who had contracted with ADHS, OPHEPR to provide bioterrorism and public health emergency preparedness services to their members. These were: Cocopah Tribe, Ft. Mohave Indian Tribe, Gila River Indian Communities, Hopi Tribe, Hualapai Tribe, Kaibab-Paiute Tribe, Navajo Nation, Salt River Pima-Maricopa Indian Community, San Carlos Apache Tribe, Tohono O'odham Nation, Tonto Apache Tribe, and White Mountain Apache Tribe. Training was open to participation by all Arizona tribes.

Tribes were invited to select other interested personnel to participate from agencies such as the Bureau of Indian Affairs or the Indian Health Service. Participating tribes were also encouraged to include personnel from tribal health and human service programs, emergency management, tribal executive offices, tribal councils, community leaders and members, and local county bioterrorism coordinators.

## Methods

The training session were presented in three modules approximately four hours in length over the course of one and one-half consecutive days. Training was delivered regionally to promote inter-jurisdictional discussion, learning and network development The five regions were: (1) Ft. Mohave Indian Tribe and Hualapai Tribe; (2) Gila River Indian Communities, Salt River Pima-Maricopa Indian Community, and Tonto Apache Tribe; (3) San Carlos Apache Tribe and White Mountain Apache Tribe; (4) Hopi Tribe, Navajo Nation and Kaibab-Paiute Tribe; and (5) Tohono O'odham Nation and Cocopah Tribe.

The contract specified that training content would build upon the themes of tribal sovereignty, respecting that each tribe has a long history of unique and valued language, culture and traditions, a commitment to community and family wellness and preparedness, and an understanding that preparedness is not a new concept for Native Americans. Each module incorporated relevant examples of public health emergencies within the tribal context. These examples were coordinated with the ADHS Tribal Liaison. ADHS, OPHEPR collaborated in the process of curriculum development and training format and assisted in refinement throughout the training sessions. Evaluation tools were developed jointly and implemented at the participant level.

The overall goals of the training project were to: (1) provide opportunities for participants to gain the basic knowledge, skills and abilities required of *all *public health professionals, as defined by the core public health emergency preparedness competencies published by the Centers for Disease Control and Prevention and; (2) understand how these concepts and principles may relate to the unique needs of tribal communities [2].

### Module content

Module 1: Description of the Roles of Public Health

Contents:

• Prevention of epidemics and spread of disease

• Prevention of injuries

• Protection against environmental hazards

• Protection of public through assurance of quality and accessible health services

• Promotion and encouragement of health behaviors

• Disaster preparedness, response and recovery assistance

• Health codes

Module 2: Bio-terrorism and How it Relates to Public Health

Contents:

• Recognizing an emergency

• Disaster versus emergency

• History of bioterrorism

• Bioterrorism and public health

• Agents of bio-terrorism

• Surveillance and disease tracking using technology and other methods [3]

• Other emergencies (chemical and radiological)

• Psychological response to emergencies

Module 3: Community Emergency Preparedness and Response

Contents:

• Incident Command System

• Emergency response plans

• Collaboration with County, State and Federal agencies

• Memorandums of understanding

• Mass prophylaxis clinics

• Cultural considerations in the tribal context

An Interagency Service Agreement between the Arizona Department of Health Services, Office of Public Health Preparedness and Emergency Response was instituted on May 1, 2005 with the University of Arizona Mel and Enid Zuckerman College of Public Health. Between May 1 and September 30, 2005 all the deliverables of the agreement were met.

### Planning and Assessment

The project team was assembled to implement the scope of work and included a College of Public instructional advisor and co-instructor, a MPH instructional specialist and co-instructor, a MPH- project development and evaluation specialist, a project coordinator and administrative support

Two interagency team meetings occurred in May 2005 to ensure all parties were clear on the project intent, scope of work and desired approaches. Input was sought from the key stakeholders to the project via an emailed survey and a telephone conference call with the Tribal Bioterrorism Coordinators. This elicited feedback on key public health concerns, ideas for case scenarios, delivery preferences and individuals who wished to be further involved. Bi-monthly team meetings were held to plan and coordinate project activities.

### Curricula Development

Three distinct four-hour modules were developed following the guidelines given for core content areas. Teresa Wall, MPH, consultant on the project, was the main curriculum specialist with assistance from W.F. Peate, MD, MPH, project principal investigator. Core competencies for public health professionals were built into the modules and learning objectives were developed for each module. A variety of federal, state and locally developed materials were drawn upon, adapted and referenced. The curricula, agenda format and evaluation tools were presented to the ADHS OPHEPR team on July 6, 2005 for input. Revisions were made based on that discussion.

### Coordination and Implementation

It was important to seek cooperation from the Tribal Bioterrorism (BT) Coordinators from the outset of the project to ensure they were involved in both identifying the participants from their tribal organizations and key partners such as Indian Health Service and local county health departments for their regional training. Early in the project a host tribe was identified for each region and dates were selected. The Project Coordinator then worked extensively with the host tribe's BT Coordinator to plan the logistics for the training in that region. This included choosing an appropriate venue, advertising the trainings, recruiting participants for partner agencies, working with the team to identify local representatives to provide a traditional blessing, participating in the sessions, providing examples of traditional ways public health had been practiced in their communities and giving an example of a public health emergency they had been confronted with and what strategies had been implemented. Venue costs were supplemented by the ADHS OPHEPR. Trainings were delivered according to a total of 122 participants across all five regions.

### Key Accomplishments

This training project was a collaborative effort between the Arizona Department of Health Services Office of Public Health Preparedness and Response, the College of Public Health and statewide tribal partners. Relationships were established quickly between project personnel and the tribal Bioterrorism Coordinators, particularly those identified from the host regional tribe. Each regional training drew upon the local expertise of the public health and emergency management systems which allowed them to be tailored to their unique public health concerns. Three comprehensive modules were developed and delivered giving an overview of public health to many diverse personnel. This was particularly useful for first responders many of whom had not been exposed to this subject matter. All participants received an introduction to public health emergency and bioterrorism preparedness and response creating a basis for common understanding. The Native American Liaison for ADHS was actively involved in developing the approach for the project, curricula and training design and supported logistics as problems arose. His participation at each of the regional trainings strengthened personal relationships and networks and indicated the importance for collaboration. The curricula incorporated native concepts of health and traditional public health practices and presented practical examples of issues facing tribal communities making it relevant and responsive to tribal needs.

### Challenges

There were several challenges to implementing the trainings in the field. These were mostly created by the very short timeline for the project due to funding restrictions. This meant there was little time to visit in the field with key stakeholders and to further encourage participation. As a result, the identification of the training participants was left almost entirely to the BT Coordinator for each tribe. Given the newness of many of these individuals to a newly created role, not all of these coordinators were well integrated into their local public health system. Some were not based within their health programs, but rather operated out of their emergency management departments. As a result several trainings had limited public health personnel participation.

Additionally the scope of the project was very broad as it encompassed all five regions within the state, and required cooperation between tribal, county and federal agency counterparts. Fragmentation within each local public health system resulted in some communication breakdowns and last minute requests.

Other challenges to participation in the training included limited resources available to the tribes. In some cases there were no travel funds for relevant personnel to attend the training session. In other instances, public health emergency preparedness and issues of bioterrorism were not considered priorities particularly compared other competing needs facing under-funded tribal health programs. Subsequently, there were several of the tribes whose BT coordinators and public health personnel were not represented at the training.

In regards to the curriculum, a "one-size fits all" approach created some challenges to meeting the needs of the audience. Due to the diverse backgrounds, roles and skills sets of the participants it was difficult to find the right pitch for all. For some it was too basic and for others too advanced. The content areas required for each module were very broad and it was difficult to present all the content comfortably in three half-day sessions.

### Evaluation Summary

The overall quality of the training series was evaluated highly. Of the 80 respondents across all five regional trainings, a 66% survey response rate, the average rating regarding overall satisfaction with the quality of the trainings was 4.2 out of 5, placing the average between the satisfied to very satisfied categories.

The most salient topics participants reported as a result of the training were :

• The importance of tribal and nontribal interdepartmental communication and collaboration

• The need for all personnel to understand the incident command system (ICS)

• The importance of emergency planning and preparation and a cohesive mutually understood approach

• The need to be vigilant

• The need to incorporate cultural and other public health competencies into emergency preparedness plans

• Better understanding of tribal infrastructure

• Need for collaboration with county, state and federal partners

• Practical information about how tribal community public health preparedness response is organized and who to call upon for assistance

• The need to include public health to a greater degree in emergency planning and response.

### Recommendations for Refinement

Refinements of the trainings came from evaluation data as well as from the project team. Key recommendations from participants included making the sessions longer with more interactive group scenarios. Others thought the training could be condensed into a one-day training with less emphasis on the history and mission of public health. Many requested more training of a similar nature to be held within their local setting with further encouragement of tribal leaders to attend.

The project team suggests that an introduction to public health could be useful on its own to tribal personnel and leaders who have limited exposure to the role of public health.

Training would most likely have drawn greater participation if conducted in the individual tribal communities with support from leadership and could have been tailored further to their unique concerns and stage of emergency preparedness.

### Future training needs

• Develop advanced modules with practical scenarios/exercises including surveillance and incorporate more simulated ICS scenarios and drills in the tribal context.

• Assist BT Coordinators in identifying their key public health partners and local assets and building their collaborative leadership skills

• Continue to draw upon local experts and shared experiences

• In addition to individualized sessions for each tribe, offer two regional trainings annually to encourage inter-tribal/agency exchange on public health emergency planning and preparedness.

## Conclusion

This was a highly successful collaboration between ADHS, the College of Public Health and the statewide tribal partners. Trainings were well received and highly rated in terms of quality and usefulness. Three distinct training curricula along with products and tools have been developed and adapted for the Arizona tribal context. Sustainability of these trainings in local tribal settings has been requested by many tribal participants. Additional training needs were identified for the tribes. The statewide tribal public health emergency preparedness network was strengthened as a result of this project.
